# Phylogenetic relationships of 52 *Eimeria* species based on COI sequences

**DOI:** 10.1080/23802359.2019.1688709

**Published:** 2019-11-12

**Authors:** Qingyue Li, Chong Wang, Zhizhong Gong, Gang Liu

**Affiliations:** School of Life Sciences, Anhui Medical University, Hefei, P. R. China

**Keywords:** Eimeria, COI, phylogenetic relationship

## Abstract

Coccidiosis is an important protozoan disease of domestic animals, which frequently presents as simultaneous infections with multiple *Eimeria* species, however the relationships of *Eimeria* species are not clear at present. In this study, we sequenced the COI of *E. tenella*, *E. mitis*, *E. anseri* isolated from wintering *Anser albifrons* feces, and also downed 49 *Eimeria* species published in Genbank. The results indicated that no phylogenetic reconstruction supported monophyly of *Eimeria* species, which is different from previous studies, *Eimeria* dispersa may have arisen via host switching from another host.

Coccidiosis is an important protozoan disease of domestic animals, also is recognized as one of the most important diseases with great economic impact on wild birds and poultry industry throughout the world (Lin et al. 2011; Ogedengbe et al. 2014; Chengat Prakashbabu et al. 2017). The disease frequently presents as simultaneous infections with multiple *Eimeria* species (Apicomplexa: Eimeriidae) (Ogedengbe et al. 2013). *Eimeria* can cause serious damage to the digestive tract of the host, resulting in malabsorption of nutrients and diarrhea, which causes decreased body weight gain, and possibly lead to death, and has been one of the biggest challenges faced by the global poultry industry (Lin et al. 2011; Chengat Prakashbabu et al. 2017; Song et al. 2017). However, the relationships of *Eimeria* species are not clear at present (Ogedengbe et al. 2013; Liu et al. 2019).

COI gene provide useful markers for investigating population genetic structures, systematics and phylogenetics of organisms, it has been extensively used as genetic markers for phylogenetic analyses at different levels (Lin et al. 2011; Liu et al. 2019). Here, we sequenced the COI of three *Eimeria* species (*E. tenella*, *E. mitis*, *E. anseri*) isolated from wintering *Anser albifrons* feces which collected in Shenjin lake (E117°0′42.55″, N30°20′30.73″), Anhui province of China in January, 2019. The DNA samples were stored at −20 °C in the Cancer Cell Biology Laboratory, School of Life Sciences, Anhui Medical University (Sample codes are AHMUP20190101-AHMUP20190103). The COI sequences have been submitted to GenBank (accession number MN586863-MN586865). We also searched all the COI genes of *Eimeria* species which published in Genbank more than 450 bp in the length to this day.

The nucleotide compositions of the 52 COI sequences for *Eimeria* are biased toward A and T, with T being the most common nucleotide and G the least common. The mean total nucleotide composition was: A, 26.1%; C, 18.5%; G, 16.9%; and T, 38.5%; the average AT content (55.6%) being slightly higher than the CG content (44.4%), which is similar to the most *Eimeria*, in which the rarest nucleotide is G (Lin et al. 2011; Ogedengbe et al. 2014; Hafeez et al. 2015; Liu et al. 2019).

Phylogenetic trees were estimated using ML and BI methods, to study the phylogeny of the *Eimeria* species. Corresponding *Isospora butcherae* (KY801687) sequence was used as outgroup. Phylogenetic analyses of 52 *Eimeria* species based on COI revealed five distinct groups with high statistical support (Figure 1). No phylogenetic reconstruction supported monophyly of *Eimeria* species, is different from previous studies, *Eimeria* dispersa may have arisen via host switching from another host (Lin et al. 2011; Chengat Prakashbabu et al. 2017; Song et al. 2017). Though the inferred phylogenetic trees in this study provided new evidence for understanding the evolution of *Eimeria* species, the present dataset has its limitation in reconstruction of the intra-generic relationships.

## Nucleotide sequence accession number

The COI sequences of *E. tenella*, *E. mitis* and *E. anseri* have been assigned with GenBank accession numbers MN586863-MN586865.

**Figure 1.**
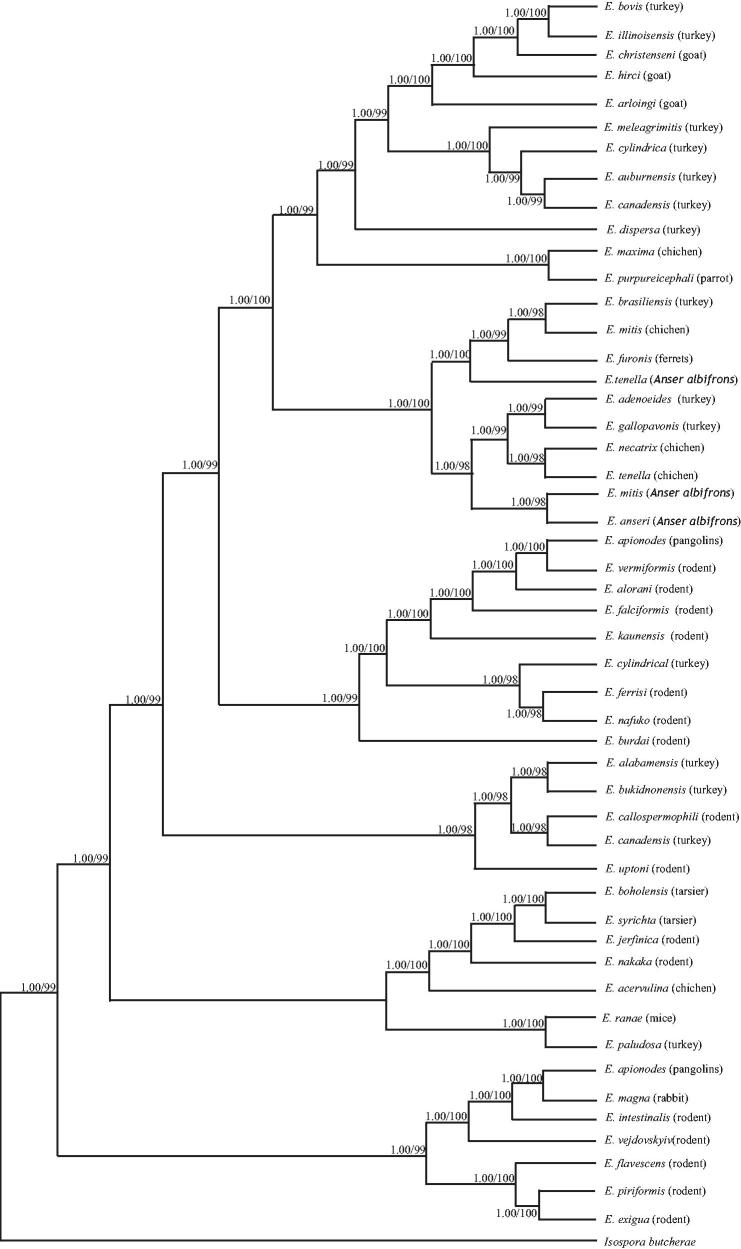
Phylogenetic trees based on COI sequeces of 52 *Eimeria* species by the ML and BI methods. Numbers at each node are bootstrap values from three analyses (Maximum likelihood/Bayesian inference). Notes: *E. acervulina*: FJ_236443, *E. meleagrimitis*: KC_346353, *E. adenoeides*: KC_346360, *E. mitis*: FR_796699, *E. alabamensis*: KU_351690, *E. nafuko*: JQ_993708, *E. alorani*: JQ_993701, *E. necatrix*: EU_025108, *E. apionodes*: JX_464221, *E. nkaka*: JQ_993697, *E. arloingi*: KX_857470, *E. paludosa*: KJ_767189, *E. auburnensis*: KU_351693, *E. piriformis*: JQ_993698, *E. boholensis*: MH_350860, *E. purpureicephali*: KU_140598, *E. bovis*: KU_351697, *E. ranae*: MH_698563, *E. brasiliensis*: KU_351698, *E. subspherica*: KU_351704, *E. bukidnonensis*: KU_351700, *E. syrichta*: MH_350859, *E. burdai*: JQ_993709, *E. tamiasciuri*: KT_184375, *E. cahirinensis*: JQ_993687, *E. tenella*: FJ_236453, *E. callospermophili*: JQ_993688, *E. uptoni*: KU_216013, *E. Canadensis*: KU_351701, *E. vejdovskyi*: JQ_993699, *E. christenseni*: KX_857468, *E. vermiformis*: MH_777577, *E. cylindrical*: KU_351702, *E. intestinalis*: JQ_993693, *E. dispersa*: HG_793048, *E. irresidua*: JQ_993694, *E. exigua*: JQ_993691, *E. ivitaensis*: MH_892075, *E. falciformis*: MH_777576, *E. jerfinica*: KU_216033, *E. ferrisi:* MH_777593, *E. kaunensis*: KU_216034, *E. flavescens*: JQ_993692, *E. magna*: JQ_993695, *E. furonis*: MF_774036, *E. maxima*: FJ_236459, *E. gallopavonis*: HG_793051, *E. illinoisensis*:KU_351703, *E. hirci*: KX_857469, *E. tenella*: MN_586863, *E. mitis*: MN_586864, *E. anseri*: MN_586865.

## References

[CIT0001] Chengat Prakashbabu B, Thenmozhi V, Limon G, Kundu K, Kumar S, Garg R, Clark EL, Srinivasa Rao AS, Raj DG, Raman M, et al. 2017 *Eimeria* species occurrence varies between geographic regions and poultry production systems and may influence parasite genetic diversity. Vet Parasitol. 23:362–372. 10.1016/j.vetpar.2016.12.003 PMC5239766 28043390

[CIT0002] Hafeez MA, Shivaramaiah S, Dorsey KM, Ogedengbe ME, El-Sherry S, Whale J, Cobean J, Barta JR 2015 Simultaneous identification and DNA barcoding of six *Eimeria* species infecting turkeys using PCR primers targeting the mitochondrial cytochrome c oxidase subunit I (mtCOI) locus. Parasitol Res. 114(5):1761–1768. 25678350 10.1007/s00436-015-4361-y

[CIT0004] Lin RQ, Qiu LL, Liu GH, Wu XY, Weng YB, Xie WQ, Hou J, Pan H, Yuan ZG, Zou FC, Hu M, et al. 2011 Characterization of the complete mitochondrial genomes of five *Eimeria* species from domestic chickens. Gene. 408:28–33. 10.1016/j.gene.2011.03.004 21402132

[CIT0005] Liu G, Li QY, Wang C, Xu CL 2019 The complete mitochondrial genome of Eimeria anseris from the wintering greater white-fronted goose in Shengjin Lake, China, and phylogenetic relationships among *Eimeria* species. Parasitol Res. 118(4):1299–1306. 30778751 10.1007/s00436-019-06252-7

[CIT0006] Ogedengbe ME, El-Sherry S, Whale J, Barta JR 2014 Complete mitochondrial genome sequences from five *Eimeria* species (Apicomplexa; Coccidia; Eimeriidae) infecting domestic turkeys. Parasit Vectors. 7:335. 25034633 10.1186/1756-3305-7-335 PMC4223602

[CIT0007] Ogedengbe ME, Hafeez MA, Barta JR 2013 Sequencing the complete mitochondrial genome of *Eimeria mitis* strain USDA 50 (Apicomplexa: Eimeriidae) suggests conserved start positions for mtCOI- and mtCOIII-coding regions. Parasitol Res. 112(12):4129–4136. 24013344 10.1007/s00436-013-3604-z

[CIT0008] Song H, Liu D, Xu J, Wu L, Dai Y, Liu M, Tao J 2017 The endogenous development and pathogenicity of *Eimeria anseris* (Kotlan, 1932) in domestic goslings. Parasitol Res. 116(1):177–183. 27770197 10.1007/s00436-016-5274-0

